# Recognition of Obesity and Perceptions of Weight Loss Management in Patients With Chronic Kidney Disease: A Retrospective Cross-Sectional Study

**DOI:** 10.1177/20543581221129465

**Published:** 2022-10-12

**Authors:** Michael Chiu, Louise Moist, Ahmed Al-Jaishi, Arsh K. Jain

**Affiliations:** 1 Division of Nephrology, Department of Medicine, Western University and London Health Sciences Centre, ON, Canada; 2 Kidney Clinical Research Unit, London Health Sciences Centre, ON, Canada; 3 Clinical Epidemiology Program, The Ottawa Hospital Research Institute, ON, Canada

**Keywords:** obesity, quality improvement, chronic kidney disease

## Abstract

**Background:**

Obesity is, directly and indirectly, linked to the progression of chronic kidney disease (CKD). However, nephrologists’ recognition of obesity and willingness to address and manage obesity are unknown.

**Objectives:**

The aim of this article is to investigate if obesity is recognized and documented in the clinical encounter and to examine nephrologists’ perceptions of obesity and comfort with weight loss management.

**Design:**

We conducted a 2-part study. Part I used a retrospective chart review and part II used an anonymous online survey of practicing nephrologists (n = 14) in our center.

**Setting:**

The study took place in the Multi-care Kidney Clinic (MCKC) at London Health Sciences Centre in London, Ontario, Canada.

**Patients:**

In part I, we conducted a retrospective chart review of 10 random patients with advanced CKD and obesity (body mass index [BMI] > 30 kg/m^2^) from each of the nephrologists between January and December 2019.

**Methods:**

In part I, charts were assessed for documentation of obesity and/or a treatment plan (lifestyle counseling, pharmacologic intervention, and specialist referral). In part II, a survey completed by the nephrologists explored their current experience and perceptions of obesity and comfort with weight loss management. Responses were ranked on a 5-point Likert scale.

**Results:**

In all, 140 patient charts were reviewed. The median age was 69 (interquartile range [IQR] = 60-77) years, estimated glomerular filtration rate (eGFR) was 17 (IQR = 12-20) ml/min/1.73 m^2^, weight was 99 (IQR = 90-116) kg, and BMI was 36 (IQR = 33-40) kg/m^2^. Obesity with a BMI was documented in 36 (26%) charts, and only 2 (1%) documented a weight loss plan, which only included non-pharmacologic strategies. There were 13 survey responses (93% response rate). All nephrologists agreed that obesity negatively affects the health of patients with CKD. Twelve (92%) reported discussing obesity with patients, but none felt that they had time to treat it. All reported discussions of obesity would evoke a negative patient response, while 5 (38%) thought patients actually want to discuss obesity. Regarding treatment, 8 (62%) nephrologists felt comfortable with non-pharmacologic treatment, but only 1 respondent was comfortable with pharmacologic treatments. Twelve (92%) nephrologists thought patients should be referred to a specialist.

**Limitations:**

There was limited generalizability as this was a single center study. The BMI may reflect hypervolemia rather than body mass.

**Conclusion:**

In our study, nephrologists rarely document and manage obesity in patients with advanced CKD, despite their perception of treatment benefits. Improved outcomes of obesity management for patients with CKD will require increased knowledge and clinical tools to efficiently address obesity with patients.

## Abrégé

**Contexte:**

L’obésité est directement et indirectement liée à la progression de l’insuffisance rénale chronique (IRC). La reconnaissance de l’obésité par les néphrologues et leur volonté de la prendre en charge demeurent toutefois mal connues.

**Objectifs:**

Vérifier si l’obésité est reconnue et documentée lors de la rencontre clinique. Examiner les perceptions des néphrologues quant à l’obésité et leur confort face à la gestion du poids.

**Conception:**

Notre étude était constituée de deux parties. La première consistait en un examen rétrospectif des dossiers et la deuxième en un sondage anonyme mené en ligne auprès des néphrologues praticiens (n = 14) de notre centre.

**Cadre:**

La clinique multidisciplinaire en santé rénale (CMSR) du London Health Sciences Centre de London, en Ontario (Canada).

**Sujets:**

Dans la première partie, nous avons examiné rétrospectivement les dossiers de dix patients aléatoires atteints d’IRC de stade avancé et jugés obèses (IMC > 30 kg/m^2^) pour chacun des néphrologues participants entre janvier et décembre 2019.

**Méthodologie:**

Dans la première partie, les dossiers ont été examinés à la recherche d’une mention de l’obésité et/ou d’un plan de traitement (conseils sur le mode de vie, intervention pharmacologique, aiguillage vers un spécialiste). Dans la deuxième partie, un sondage réalisé auprès des néphrologues a exploré leur expérience et leurs perceptions actuelles sur l’obésité et leur confort quant à la gestion de la perte de poids. Les réponses ont été classées sur une échelle Likert de 5 points.

**Résultats:**

En tout, 140 dossiers patients ont été examinés. L’âge médian s’établissait à 69 ans (ÉIQ: 60-77), le DFGe médian à 17 ml/min/1,73 m^2^ (ÉIQ: 12-20), le poids médian à 99 kg (ÉIQ: 90-116) et l’IMC médian à 36 kg/m^2^ (ÉIQ: 33-40). L’obésité avec IMC était documentée dans 36 dossiers (26%) et seulement deux dossiers (1%) comportaient un plan de perte de poids, lequel ne comprenait que des stratégies non pharmacologiques. Treize néphrologues ont répondu au sondage (taux de réponse: 93%). Tous ont convenu que l’obésité affecte négativement la santé des patients atteints d’IRC; douze (92%) ont mentionné discuter d’obésité avec leurs patients, mais aucun ne pensait avoir le temps de la traiter. Tous les répondants ont indiqué que les discussions abordant l’obésité recevaient un accueil négatif de la part du patient; seuls 5 néphrologues (38%) étaient d’avis que les patients sont réellement ouverts à discuter d’obésité. Quant à son traitement, huit néphrologues (62%) se sont dits à l’aise avec un traitement non pharmacologique; un seul répondant était à l’aise avec les traitements pharmacologiques. Selon douze répondants (92%), les patients devraient être dirigés vers un spécialiste.

**Limites:**

Étude dans un seul centre, ce qui limite la généralisabilité. L’IMC pourrait refléter une hypervolémie plutôt que la masse corporelle.

**Conclusion:**

Dans notre étude, les néphrologues ont rarement documenté et pris en charge l’obésité de leurs patients atteints d’IRC de stade avancé, et ce, bien qu’ils croient aux avantages de son traitement. Pour améliorer la prise en charge de l’obésité chez les patients atteints d’IRC, il faudra améliorer les connaissances et les outils cliniques permettant de la traiter efficacement chez ces patients.

## Introduction

Obesity has reached epidemic proportions globally. The World Health Organization approximates that 650 million people worldwide are obese.^1,2^ In addition, over 50% of patients with chronic kidney disease (CKD) also suffer from obesity in the United States.^3^ There is mounting evidence that obesity, directly and indirectly, contributes to worsening kidney function^4^ and the development of end-stage kidney disease.^5^ Furthermore, it is often a barrier to kidney transplantation as obesity contributes to up to 30% of exclusions from kidney transplants.^6^ Despite the deleterious effects of obesity on kidney function, it is unknown if nephrologists recognize or address obesity management in patients with CKD and obesity.

Several studies have demonstrated that there are hormonal and metabolic imbalances that prevent patients from losing weight and maintaining weight loss.^7^ This has created a paradigm to actively treat obesity as a chronic medical condition beyond only using lifestyle modifications such as diet, exercise, and cognitive behavioral therapy. Advances in treatment include pharmacologic interventions such as glucagon-like peptide-1 receptor agonists (GLP-1 RA). These medications have shown significant weight loss compared to placebo.^8,9^ Studies on GLP-1 RAs demonstrate that only using non-pharmacologic treatment, such as diet and exercise, results in rebound weight gain after 12 weeks of therapy.^10,11^ This further suggests that bariatric interventions are necessary to induce sustained weight loss.

Obesity is often managed by primary care or endocrinologists.^12,13^ It is defined as a body mass index (BMI) ≥ 30 kg/m^2^. The BMI is a simple screening tool that estimates body fat in CKD and dialysis patients.^14 [Bibr bibr12-20543581221129465],15^ Endocrinology guidelines recommend acknowledging obesity by documenting BMI and implementing a weight reduction strategy.^12^ Effective strategies include a referral to a dietician, lifestyle modification such as exercise counseling, pharmacologic therapy, and a referral to bariatric surgery. These interventions have been shown in observational studies to reduce proteinuria and may even improve kidney function.^16^ Despite the advances in obesity management, published guidelines in obesity do not specifically address management in patients with CKD.^12,13^ Therefore, nephrologists may not consider it their responsibility to recognize and or manage obesity. This creates a clinical care gap in how patients with obesity and CKD are managed.

This study aims to assess if nephrologists routinely document BMI and address weight loss in patients with advanced CKD and obesity. Second, this study will investigate nephrologists’ perceptions of obesity in their patients and their comfort in managing weight loss. Finally, the study will help identify what nephrologists believe are the barriers to managing obesity in patients with CKD. The study results may support the need for quality improvement interventions to ensure comprehensive care for the growing population of patients with CKD and obesity.

## Materials and Methods

This was a 2-part study. In part I, we conducted a retrospective cross-sectional study of patients with obesity followed in a multidisciplinary advanced CKD clinic at London Health Sciences Centre (LHSC) in London, Ontario, Canada. This clinic provides specialized care to almost 1300 patients who have access to professionals such as nephrologists, social workers, dieticians, and pharmacists that provide comprehensive care to preserve kidney function and manage the complications of kidney disease. These patients are transitioned to this care model by their nephrologist with criteria including a risk of requiring dialysis of at least 10% in 2 years as calculated by the Kidney Failure Risk Equation^17^ or an estimated glomerular filtration rate (eGFR) of <15 ml/min/1.73m.^2^ In part II, an anonymous online survey of nephrologists at LHSC was performed to investigate their experience with treating obesity and their willingness and comfort to manage weight loss for their patients.

### Patient Selection for Chart Review

A convenience sample of 10 patients over 18 years of age with a BMI greater than or equal to 30 kg/m^2^ from 14 nephrologists were randomly selected using a random number generator for audit between January 2019 and December 2019. Patients were only included once, and all patients were unique to each nephrologist. Patients with a history of bariatric surgery or those who are enrolled in a weight management program were excluded. In addition, patients choosing conservative care or those with a history of dementia were excluded.

### Chart Review

All patient encounters and clinical data are documented in an electronic medical record, including patient demographics, weight, height, and clinical notes. Height and weight are recorded at the first encounter in the nephrology clinic, and the weight is updated on subsequent encounters. The electronic medical record (EMR) automatically calculates the BMI once the weight (kg) and height (m) are recorded. The current medication list and laboratory data are documented at each clinical encounter. If the data were not documented, a previous note within 6 months was used to fill in missing information on medications or lab values. If it was not documented within 6 months, the information was recorded as “not documented.” The clinical note was used to assess documentation of BMI and a weight loss strategy for patients. Strategies could include lifestyle modifications (diet, exercise, and cognitive behavioral therapy), pharmacologic intervention (use of GLP-1 RAs), or referral to a specialist for obesity management.

Characteristics of the 14 nephrologists were collected, including years of practice and the use of electronic documentation versus dictation for creation of the clinical note. The type of documentation used was collected as it was thought that electronic documentation might be associated with an increased likelihood of BMI documentation as it can be easily pulled from the EMR into the clinical note.

### Statistical Analysis

All continuous variables are summarized as a median with an interquartile range (IQR). Categorical variables are described as a frequency as a percentage. The BMI was included as a continuous variable. We fitted a multivariable generalized estimating equation (GEE) model with a binomial distribution and logit link taking into account correlated BMI documentation (primary outcome) at the nephrologist level. The model included patient characteristics, such as age, sex, diabetes, and BMI, and physician characteristics, including years of independent practice and use of electronic documentation. Diabetes was included as a covariate as, of all of the baseline variables that were collected, it is most highly associated with obesity.^18^ We used SAS version 9.4 (SAS Institute Inc, Cary, North Carolina) for all analyses. *P* values below an alpha of .05 were considered statistically significant.

### Survey of Nephrologists

The survey was completed between February and March 2021. The 14 nephrologists were individually emailed a link to the anonymous online survey, which used the Alchemer platform (previously known as SurveyGizmo). This survey was created by nephrologists who have a strong interest in obesity management. Participation was voluntary and without incentivization. The survey was created to explore various themes, including nephrologists’ knowledge of the effects of obesity on kidney function, perceptions of patients’ understanding of obesity, recent experience in addressing obesity, desire for education on obesity, perception of their responsibility to treat obesity, and finally their comfort in treating obesity. Responses were ranked on a 5-point Likert scale between *strongly disagree* to *strongly agree*. This survey was created by the authors (M.C., L.M., and A.J.) after a literature review of already published obesity surveys.

This study was conducted with approval from the Research Ethics Board at The University of Western Ontario (REB reference ID 116525).

## Results

### Part I: Chart Review

The patient characteristics are presented in Table 1. The median (IQR) age of patients was 69 (60-77) years, 46% were female, the median eGFR was 17 (12-20) ml/min/1.73 m^2^, the median weight was 99.3 (90.1-115.8) kg, and the median BMI was 35.8 (32.6-39.9) kg/m^2^. Thirty-six of 140 (26%) patients had a documented BMI in their clinical notes. Two patients had both BMI documentation and a weight loss strategy. The weight loss plans included lifestyle modifications such as caloric restriction and increased physical activity in these encounters. There was no documentation of a pharmacologic intervention or referral to a weight management program.

**Table 1.** Patient Characteristics.

	Number (n = 140)
Demographics	
Age (years), (IQR)	69 (60-77)
Female	50 (36%)
Anthropometric measurements	
Weight (kg), (IQR)	99.3 (90.1-115.8)
Body mass index (kg/m^2^), (IQR)	35.8 (32.6-39.9)
30-34.4	60 (43%)
35-39.9	46 (33%)
40-44.9	24 (17%)
45+	10 (7%)
Laboratory measurements	
eGFR (IQR)	17 (12-20)
Albumin to creatinine ratio (IQR)	61 (18.3-220.1)
Hemoglobin A1C (IQR)	7.0 (5.9-8.1)
Etiology of kidney disease	
Diabetes	79 (56%)
Hypertension	54 (39%)
Renovascular disease	6 (4%)
NSAIDS	4 (3%)
Glomerulonephritis	11 (8%)
Other	48 (34%)
Etiology not documented	2 (1%)
Obesity-related comorbidities	
Diabetes	93 (66%)
Hypertension	98 (70%)
Dyslipidemia	23 (16%)
Cardiovascular disease	37 (26%)
Obstructive sleep apnea	8 (6%)
Osteoarthritis	13 (9%)
Gastroesophageal reflux disease	6 (4%)
Depression	7 (5%)
Comorbidities not documented	5 (4%)
Medications	
ACE inhibitor or angiotensin receptor blocker	67 (48%)
Alpha blocker	32 (23%)
Beta blocker	82 (59%)
Calcium channel blocker	74 (53%)
Diuretic	100 (71%)
Glucagon-like peptide-1 receptor agonist	1 (1%)
SGLT2 inhibitor	0
Not documented	12 (9%)

IQR = interquartile range; eGFR = estimated glomerular filtration rate; NSAIDs = non-steroidal anti-inflammatory drugs; ACE = angiotensin-converting enzyme; SGLT2 = sodium-glucose co-transporter-2.

There was wide variability between nephrologists’ practices. Six (43%) nephrologists did not document BMI at all, 4 (29%) documented BMI in 1 to 2 patients out of 10, and the other 4 (29%) documented BMI in 3 or more patients. The results of the multivariable GEE model are described in Tables 2 and 3. Males had a higher odds of BMI documentation (odds ratio [OR] = 2.76, 95% confidence interval [CI] = 0.99-7.74), though this was not statistically significant. The use of an electronically documented clinic note was also associated with increased odds of documenting the BMI compared to a dictated clinical note (OR = 11.73, 95% CI = 1.00-137.87). Documenting BMI was lower in nephrologists with more years in practice compared to those with fewer years (OR = 0.83, 95% CI = 0.72-0.96).

**Table 2.** Covariates Associated With BMI Documentation in Patients With Obesity.

Covariates	Point estimate	95% confidence interval		*P* value
		Confidence limits		
Age (years)	1.01	0.95	1.06	.82
Diabetes	2.79	0.65	12.02	.17
Sex (male)	2.76	0.99	7.74	.05
BMI	1.05	0.98	1.12	.17
Electronic documentation	11.73	1.00	137.87	.05
Nephrologist years in practice	0.83	0.72	0.96	.01

BMI = body mass index.

**Table 3.** Frequency of BMI Documentation by Covariate in the General Estimating Equation Model.

Covariates	BMI	
	Not documented (n = 104)	Documented (n = 36)
Age (years), median (IQR)	69 (59-76)	69.5 (60-78)
Diabetes (%)	67 (64%)	27 (75%)
Sex (male)	64 (62%)	26 (72%)
BMI, median (IQR)	35.5 (32.7-39.8)	37.4 (32.1-40.8)
Electronic documentation (%)	55 (53%)	35 (97%)
Nephrologist years in practice, median (IQR)	22 (17-29)	12 (8-12)

BMI = body mass index; IQR = interquartile range.

### Part II: Nephrologists’ Survey

We surveyed 14 practicing nephrologists in the clinic with a 93% response rate. Responses for agreeing and strongly agreeing have been combined for this report. The results of the survey can be found in Table 4.

**Table 4.** Survey Responses.

Question	% agree/strongly agree
Nephrologists’ attitude toward the effect of obesity on CKD	
1. Obesity contributes to the progression of CKD	100
2. Obesity has negative health implications for CKD patients	100
3. Obesity is a chronic disease that has effective treatments	69
Nephrologists’ perception of patients’ understanding of obesity	
1. Patients understand that obesity is a chronic disease	39
2. Patients understand that obesity has effective treatments	0
Nephrologists’ experience addressing obesity	
1. I talk to my patients about obesity	85
2. I motivate my patients to lose weight	85
3. Patients want me to talk about obesity	8
4. Discussing obesity with the patient can evoke negative emotions/experiences	92
Educating patients	
1. Patients want educational materials on treatments for obesity	39
2. Patients understand that their weight may be a barrier for kidney transplant	62
3. Educational materials for weight loss would improve care for patients potentially eligible for transplant	69
Educating nephrologists	
1. I want education on available treatments for obesity	100
2. I want to educate patients on obesity	85
Nephrologists’ desire to treat obesity	
1. It is important for nephrologists to talk to patients about obesity	92
2. It is important for nephrologists to treat obesity	62
3. I have time to treat obesity	0
4. I am comfortable counseling on the non-pharmacologic treatments for obesity	54
5. I am comfortable treating with the pharmacologic treatments for obesity	8
6. Patients with obesity should be referred to a specialist to treat obesity	85

CKD = chronic kidney disease.

All nephrologists responded that they either agree or fully agree that obesity negatively impacts patients with CKD. All nephrologists agreed that obesity contributes to CKD progression and that obesity also results in other poor health implications for their patients. Nephrologists also shared a desire to treat obesity, as 12 (85%) reported discussing obesity with their patients and encouraging weight loss. Twelve (85%) nephrologists also wanted to educate their patients on obesity. In addition, they all shared a desire to learn about available treatments for obesity. Some potential barriers to the treatment of obesity include time constraints (none felt they had time to treat obesity) and their perceived ability to treat obesity. Regarding the nephrologists’ ability to treat obesity, 8 (57%) felt comfortable counseling on non-pharmacologic treatments for obesity, and one (8%) nephrologist was comfortable prescribing pharmacologic treatments for obesity.

## Discussion

The chart review results are not surprising and suggest that obesity is poorly recognized in an outpatient multidisciplinary clinic for patients with advanced CKD. Furthermore, in patients with obesity, nephrologists rarely discuss a weight loss strategy. Only 25% of encounters had the patient’s BMI documented, and only 1% of encounters documented a weight loss strategy. The survey attempted to elucidate the barriers that nephrologists face in addressing obesity and managing weight loss in their patients. Based on the survey results, a lack of time to treat obesity and a lack of knowledge and comfort in using medical weight loss treatments are the main impediments for nephrologists to treat this clinical issue. However, there is a discrepancy between the chart review results and the survey responses. Although most nephrologists report talking to their patients about obesity and encouraging weight loss, this was rarely documented, and BMI was documented infrequently.

This study attempted to identify factors that may increase the odds of documenting the BMI in patients with obesity. The model suggests that electronic documentation may help document BMI as the results can be recognized and pulled directly from the EMR. In addition, younger nephrologists are more likely to document BMI. This association does not distinguish if younger nephrologists are more likely to use electronic documentation where they can “copy & paste” the BMI into their note or if they are more likely to recognize obesity as a clinical issue. However, the model results suggest that using electronic documentation alone is insufficient to increase BMI documentation because some nephrologists used electronic documentation but still did not document BMI, which explains the wide confidence interval. Therefore, obesity seems to be more recognized in younger nephrologists who also use electronic documentation.

Interestingly, male patients had higher odds of having their BMI documented than females. Previous literature demonstrates that females experience more weight discrimination than males.^19^ In the study, discrimination was defined as either “being treated poorly due to one’s weight” or as “unequal treatment” due to one’s weight. Our study suggests that these biases must also be overcome by nephrologists as female patients with CKD may not be receiving the same recognition or treatment for obesity. This may be due to nephrologists offering different care based on sex or that nephrologists are less comfortable addressing weight to female patients. Further investigation to distinguish the difference is necessary.

This is the first study to investigate nephrologists’ attitude toward obesity management and directly relate it to how patients with advanced CKD are treated in an outpatient nephrology clinic. The chart review assessed a large number of patients with advanced CKD who are at high risk of requiring dialysis. We also captured important clinical data, including medication use, comorbidities, and laboratory data. Furthermore, our survey had a 93% response rate, which suggests high motivation of the nephrologists at this center to enact change.

This study has limitations. First, this is a single-center study, which limits generalizability. However, it highlights the need for nephrologists to audit their practice to identify if they are providing the most comprehensive care for their patients. In addition, poor clinical documentation is common and affects the quality of care patients receive.^20^ Inaccurate documentation occurs throughout multiple specialties.^21-23^ Thus, although physicians did not document BMI or discuss obesity in the chart review, they may have addressed obesity directly with the patient and failed to document it. In addition, some notes did not contain up-to-date, pertinent information, including medications or laboratory values. Another limitation is that this study used BMI as the sole marker for obesity; unfortunately, BMI may be affected by other clinical issues such as hypervolemia. Thus, some included patients may be volume overloaded rather than obese, which is amenable to diuresis. While there are other ways to measure obesity (ie, waist-to-hip ratio, waist circumference), it is most often used and readily available.^12^ Other anthropometric measurements for obesity are not captured in this clinic. Finally, there are no validated surveys that assess nephrologists’ perspectives on obesity or their comfort in managing weight loss. We developed this survey to explore themes that we thought would be important to assess potential barriers to obesity management.

The results of our study are consistent with other studies in primary care and general internal medicine clinics. Obesity is poorly documented.^23,24^ However, if we compare our results to the published literature, nephrologists appear less likely to document obesity management than other specialties. In a general internal medicine clinic, weight loss strategy was documented 20% of the time, and in a primary care clinic, it was documented 5% of the time. This may be because the previous studies were located in the United States, where obesity management is a quality indicator according to the National Quality Foundation, and there may be incentivization for documentation and treatment. The previous studies were also done in more generalized specialties, which provide more comprehensive care. Our survey helps elucidate this discrepancy as nephrologists blame time constraints and are not comfortable addressing obesity due to previous negative experiences and unfamiliarity with obesity treatments. These are potential targets for interventions to improve obesity management.

The discrepancy between nephrologists’ intentions to treat obesity and manage obesity can be addressed using implementation science frameworks. One such framework is the Behavior Change Wheel (BCW), as published by Michie et al,^25^ used effectively in previous quality improvement projects.^26^ The framework suggests that behavior is affected by capability, opportunity, and motivation. Capability is the individual’s capacity to engage in the behavior. Opportunity entails the outside factors influencing the provider to engage in the behavior. Motivation includes all the processes that empower the provider to engage in the behavior. Based on our survey, nephrologists have the motivation to address obesity as 85% of nephrologists want to educate their patients on obesity and 100% want education on available treatments for obesity. Therefore, efforts must be focused on improving the capability and opportunity of nephrologists to manage obesity.

Capability can be increased by educating nephrologists about pharmacologic treatments for obesity and training how to effectively discuss obesity with patients in an open and non-judgmental manner. Pharmacologic treatment of obesity in CKD patients with diabetes includes using GLP-1 RAs, which KDIGO endorses as the recommended second-line treatment for CKD patients with obesity and diabetic kidney disease.^27^ Importantly, as most patients (66%) included in this study had diabetes, an intervention to increase GLP-1 RA use in this specific population is warranted. Future quality improvement studies should be done on how to best implement this intervention, which may be center or nephrologist dependent. Merely addressing obesity with patients appears to be a significant barrier as 92% of nephrologists are worried that discussing obesity can evoke negative emotions. Many frameworks to overcome this challenge exist. One example is called the “6A Framework,” which provides a word-by-word script to engage with the patient and develop a shared decision-making weight loss plan.^28^

Opportunity can be addressed by creating aids to help providers access anthropometric data on the electronic medical record (BMI flag) and create a streamlined referral process to a weight management program. A quality improvement intervention by Wang’ondu et al^29^ demonstrated that educating providers on how to access weight and BMI from the medical record quickly increased documentation of obesity from 46% to 79%. While this intervention increased the rate of BMI documentation, it did not necessarily increase the likelihood of referrals to weight management services.^30^ Based on our survey, lack of time, knowledge of weight loss, and comfort with discussion about weight loss are barriers to weight loss management. Several nephrologists also support a referral process to a weight management program for ongoing care. Therefore, developing a streamlined referral process to a medically supervised weight management program may help overcome the aforementioned barriers and increase treatment of obesity in patients with CKD.

This project is the beginning of a program to improve obesity care for patients with CKD. It is an important issue that directly and indirectly affects this patient population. The results of this study serve as part of a root cause analysis to initiate a series of quality improvement interventions to enact sustainable changes in obesity management for nephrology.

## Conclusions

Obesity is poorly documented, and a weight loss plan is rarely implemented in patients with CKD and obesity. The survey in this study demonstrates that nephrologists are motivated to address weight loss, yet this does not translate into practice. Future studies are needed to study the effectiveness of interventions to increase the recognition of obesity and increase weight loss management in patients with CKD. As obesity has significant health implications for patients with CKD, nephrologists should play a role in its management.
